# Germline Mutations and Polymorphisms in the Origins of Cancers in Women

**DOI:** 10.1155/2010/297671

**Published:** 2010-01-10

**Authors:** Kim M. Hirshfield, Timothy R. Rebbeck, Arnold J. Levine

**Affiliations:** ^1^The Cancer Institute of New Jersey, University of Medicine and Dentistry of New Jersey, New Brunswick, NJ 08901, USA; ^2^Department of Biostatistics and Epidemiology, University of Pennsylvania School of Medicine, Philadelphia, PA 19104, USA; ^3^Institute for Advanced Study, Princeton, NJ 08540, USA

## Abstract

Several female malignancies including breast, ovarian, and endometrial cancers can be characterized based on known somatic and germline mutations. Initiation and propagation of tumors reflect underlying genomic alterations such as mutations, polymorphisms, and copy number variations found in genes of multiple cellular pathways. The contributions of any single genetic variation or mutation in a population depend on its frequency and penetrance as well as tissue-specific functionality. Genome wide association studies, fluorescence in situ hybridization, comparative genomic hybridization, and candidate gene studies have enumerated genetic contributors to cancers in women. These include p53, BRCA1, BRCA2, STK11, PTEN, CHEK2, ATM, BRIP1, PALB2, FGFR2, TGFB1, MDM2, MDM4 as well as several other chromosomal loci. Based on the heterogeneity within a specific tumor type, a combination of genomic alterations defines the cancer subtype, biologic behavior, and in some cases, response to therapeutics. Consideration of tumor heterogeneity is therefore important in the critical analysis of gene associations in cancer.

## 1. Inherited Mutations that Predispose to Cancers in Women

There is strong evidence that inherited genetic factors (mutations plus single nucleotide polymorphisms) can play a major role in breast cancer susceptibility [1]. Inherited mutations in a small number of genes account for about five to ten percent of women's cancers.These inherited variations, identified in breast, ovarian, and endometrial cancer susceptibility, can be characterized in the general population by their frequency and the magnitude of their impact upon a patient (Table 1).Some inherited variants occur rarely in the general population, but confer large risks to the individual. Examples of these genes are BRCA1 and BRCA2 in breast and ovarian cancers. A second class of inherited variants confers a lower risk, and these variants are also rare in the general population. An example of this class of genes is a mutation in the CHEK2 gene in breast cancer. The third class, composed of high-risk variants that are also common in the population, has never been identified by the methods presently available and may in fact not exist because it may well be strongly selected against in populations. Finally, a fourth class of inherited variants includes those that confer low disease risk to the individual, but occur at higher frequencies in populations. These include some of the recent findings from genome-wide association studies (GWASs) mostly with breast cancers. A summary of the major findings to date for these genes is in Table 1 and is discussed in what follows.

Despite these advances made in identifying inherited breast cancer susceptibility genes, the vast majority of breast cancers are sporadic, that is, no identifiable mutation in one of the known breast cancer susceptibility genes. While this may reflect the fact that we have yet to identify the next BRCA gene, it may also reflect the polygenic nature of breast cancer susceptibility. Other contributors to genetic susceptibility, for example, polymorphisms, may have a higher relative contribution to risk, but their lower penetrance makes identification more difficult. Furthermore, modification of genetic susceptibility by environmental factors, both endogenous and exogenous, may alter the degree of penetrance. Supporters of the polygenic nature of breast cancer suggest that the contributions from polymorphisms are very important because of their high frequency in the population. 

### 1.1. High-Penetrance, Low-Frequency Inherited Variants

Although inherited mutations in a small number of genes account for only about five to ten percent of women's cancers, by far the BRCA1 and BRCA2 gene mutations are the most common examples of this observation (50–70% of familial breast cancers) [2]. In some populations BRCA1 and BRCA2 mutations can account for ten percent of all breast cancers (Ashkenazi Jewish populations) and ovarian cancers but in many ethnic groups and in all populations taken together these mutations are much rarer (reviewed in [3]). The BRCA1 and BRCA2 proteins appear to be scaffolding proteins that assemble DNA repair complexes of proteins at double-strand DNA breaks (mediating homologous DNA repair processes) (reviewed in [4]). Mutations in these genes result in a faulty repair process and a high mutation rate, especially during DNA replication, leading to cancers. The penetrance of these mutations for cancer occurrence and the age of onset of these cancers in women can be quite variable. There have been a number of other possible functions ascribed to the BRCA1 and BRCA2 proteins such as ubiquitin ligase activity and a modifier of transcription and it is certainly possible that these protein complexes act in several ways [5]. Breast cancers initiated in women who are heterozygous for BRCA1 or BRCA2 often have a reduction to homozygosity at the BRCA-locus eliminating its functions. This results in DNA damage in the tumor which should activate the p53 protein resulting in apoptosis, senescence, or cell cycle arrest. If this is the case, the p53 gene product would be a suppressor of this cancer phenotype and contribute to the variable penetrance of these breast cancer genes. Consistent with this is the observation that BRCA1/2-initiated breast cancers have very high rates (29–84%) of somatic p53 mutations compared to 14–35% in non-BRCA1/2-related breast cancer [6]. 

 Inherited mutations in several other genes, such as PTEN and p53, can give rise to cancers in women. Cowden's Disease is a heterozygous deficiency in the PTEN gene that can result in breast, endometrial, and other cancers [3, 7]. The PTEN protein is a lipid (PIP-3) phosphatase that modulates a growth factor pathway, in turn regulating metabolic pathways in cells, angiogenesis, mitochondrial functions and apoptotic functions [8]. Genetic alterations in this pathway are among the most common somatic mutations observed in breast and endometrial cancers [9, 10]. Mutations in LKB1 also predispose to breast and ovarian cancers as one of the phenotypes in Peutz-Jeghers syndrome [3, 11]. Inherited defects in one allele of the p53 gene give rise to Li-Fraumeni syndrome, where a subset of the cancers observed at an early age are breast cancers [12]. 

### 1.2. Low-Penetrance, Low-Frequency Inherited Variants

This class of inherited variants is difficult to detect with existing methods because the rarity of these variants and coupled with small effect sizes this means that most association studies will not be able to detect them due to limitations in population sizes under study. In the extreme, these variants may represent “private” mutations that confer a small degree of risk to very few individuals in this population, such that nearly every person would have a unique set of predisposing alleles. While it has been difficult to detect inherited variants of this type there are several examples of this type of variant which were uncovered by examining candidate genes that an investigator suspected played a role in a cancer. Inherited alterations in the CHEK2 gene which normally produces a protein kinase found in signal transduction pathways (p53 pathway and others), alerts the cell that there is DNA damage and its loss can have an impact upon several types of cancer [13]. Similarly the ATM protein kinase harbors genetic variants that detect single- and double-strand breaks in the DNA and signals to the p53 pathway and other DNA repair processes. Variants in this gene could lower or raise the sensitivity of this DNA damage detector and impact upon the efficiency of p53 and its tumor suppressor pathway and can predispose women to breast cancers [14]. The BRIP1 gene (BRCA1 interacting protein-1) encodes a protein that is a DNA/RNA helicase of the REC Q family that binds to the carboxy-terminus of BRCA1 protein conferring an activity involved in DNA repair and variants of this gene can predispose to breast cancers [15]. Interestingly this gene product is also a component of the Fanconi anemia gene pathway for DNA repair processes. Finally the PALB2 gene product (partner and localizer of BRCA2) is part of the BRCA2 protein complex and plays a role in DNA repair. It has recently been shown to be a genetic determinant of familial breast and other cancers primarily in the certain populations, but found at even lower frequency in other populations [16].

### 1.3. Low-Penetrance, High-Frequency Inherited Variants

Fewer than 10% of breast cancers are attributable to known mutations in breast cancer susceptibility genes BRCA1 and BRCA2. The multigenic susceptibility due to common, low-penetrance risk markers is yet to be defined [1, 17–20]. Both candidate gene [21] and genome-wide association studies have identified novel markers for susceptibility [22–25] and prognosis [26]. Genome-wide association studies have become widely used to identify commonly occurring alleles at disease susceptibility loci. These studies use a large number of high-density markers to identify associations with disease that rely upon patterns of linkage disequilibrium in the human genome. GWASs have been successful in identifying genes for breast cancer, and GWASs for ovarian and endometrial cancers are underway although several investigators have validated findings from GWAS studies designed originally for breast cancer studies but employed for ovarian cancer [27]. Some of the more reproducible genes that GWASs studies have indicated can play a role in the risk for developing breast cancers include FGFR2, LSP1, MAP3K1, TGFB1, TOX3, 2q35, and 8q [17, 22, 24].

## 2. Somatic Mutations That Are Commonly Observed in Women's Cancers

Both gene amplifications and deletions can lead to common somatic mutations in women's cancers. Among the amplifications are the following. (1) HER-2/Neu, amplified in about 15% of the breast cancers, is a growth receptor that activates the Ras-MEK and the PI3K pathways in cancer cells [28]. (2) Cyclin D, amplified in about 10–12% of the breast cancers, is a subunit of the cyclin dependent kinase −4/6 that acts upon the Rb protein freeing the E2F transcription factor for entry into the cell cycle [28, 29]. (3) WIP1, amplified in about 13% of breast cancers, is a serine/threonine phosphatase that inactivates the ATM kinase and the p53 protein [30]. The GASC1 gene, which produces a histone demethylase activity, is amplified in about 5–10% of breast cancers but 20–25% of the basal breast cancers. This enzyme removes dimethyl and trimethyl groups from histone H-3 lysine-9 and 36 residues which results in altered transcriptional patterns in these cells. Inactivation of gene functions by deletion or other mechanisms commonly occurs in (1) PTEN in breast, ovarian, and endometrial cancers, and (2) p53 in HER2/neu positive breast cancers, triple negative breast cancers, and BRCA-associated breast and ovarian cancers. PI3K amplifications and activating mutations are common in breast and endometrial cancers [31, 32] and Ras activating mutations are common in endometrial cancers. Several genes such as AKT and STAT3 are often expressed at high activities in all of these cancers but without detectable amplifications of those genes. Epigenetic alterations, such as methylation of cytosine residues in CpG dinucleotides, can bring about the inactivation of genes (p16 gene in breast cancers) while mismatch repair defects have been observed to enhance the mutation rate of many genes in endometrial cancers. In addition to those somatic mutations discussed here, a large number of mutations in many oncogenes and tumor suppressor genes have been observed at lower rates in women's cancers. 

Large copy number variations in genetic loci from tumor tissues have been observed using fluorescent in situ hybridization (FISH), comparative genomic hybridization (CGH), and a reduced heterozygosity of single nucleotide polymorphisms over large regions of a chromosome. This type of genomic instability has been observed at many loci in all chromosomes in some breast tumors. Other breast tumors demonstrate little or no genomic instability (below the level of detection). As a generalization those individuals who have tumors that demonstrate very high levels of genomic instability have a poorer prognosis [33]. While some loci are repeatedly amplified, as occurs in Her2 overexpressing breast cancers, or deleted, as with PTEN in endometrial cancers, the heterogeneity of mutations in women's cancers is striking. There are many mutational paths to initiate and propagate a tumor. 

Notably however, somatic mutations often occur in genes where germline mutations in those same genes are the etiologic factors in cancer susceptibility syndromes. Alternatively, somatic mutations occur in other genes involved in regulatory aspects of those vital pathways. Despite the number of mutational pathways to initiate and propagate tumors, several specific genomic alterations are associated with particular breast cancer phenotypes. These phenotypes are manifested in their molecular profile, biology, and prognosis. Patterns of transcriptional profiles obtained from breast tumors have permitted a fairly reproducible classification of breast cancers that are derived from different cell types or have evolved under the influence of different gene expression patterns [34–38]. These different transcriptional patterns correlate well with critical diagnostic criteria (ER+, PR+, HER-2/neu+, triple negative, BRCA1) that guide both diagnosis and treatment protocols for these types of breast cancers. The classification also correlates well with some mutations such as p53, but other causal mutations such as cyclin D and WIP1 amplifications, PI3K and STAT3 activations need to be explored. Classification based upon transcriptional profiles also associates well with several clinical parameters. For example, luminal A cancers are hormone receptor positive, are diagnosed primarily in older women, are low grade with low proliferative index, and have mainly wildtype p53 [35, 38]. Luminal B cancers also tend to retain wildtype p53 but have reduced or absent expression of progesterone receptor and are more likely to recur than luminal A cancers [35, 36, 38]. In contrast to luminal tumors, basal cancers are hormone receptor negative and Her2 negative, are more likely to be diagnosed in young, premenopausal women, are high grade with high proliferative index, and are associated with higher risk of recurrence [35–38]. Her2-amplified breast cancers, regardless of hormone receptor status, are of higher grade and proliferative index, have worse prognosis with higher recurrences in first five years after diagnosis, and commonly have p53 mutations [35, 36]. Like basal tumors, BRCA1-associated breast cancers predominantly occur in young, premenopausal women, are primarily hormone receptor negative, and the most likely to carry p53 mutations [34]. Unfortunately this type of detail and analysis does not yet exist for ovarian and endometrial cancers. 

Thus, it is now clear that there are at least five types of breast cancer with characteristic transcriptional profiles that can harbor some subset of mutations that drive these cancers [39]. Importantly, each type of breast cancer calls for different treatment protocols and often results in different outcomes. We have only partially established the critical mutational patterns in each type of breast cancer and we have only begun to extend this type of analysis to other women's cancers. However, it is apparent that breast cancer heterogeneity reflects underlying genomic alterations leading to different biology and phenotypes.

## 3. Single Nucleotide Polymorphisms (SNPs) and Their Phenotypes

Inherited mutations in genes involved in DNA repair processes (BRCA1, BRCA2), cell cycle checkpoints and apoptosis (p53, Rb), and gene products that regulate critical pathways (PTEN) clearly play a central role in predetermining the initiation of cancers, often with an incomplete penetrance. Polymorphic alleles in many additional genes, often in these same signal transduction pathways, can also contribute, albeit in a smaller quantitative fashion, to the origins of a cancer, the propagation of a cancer, and the treatment responses of a cancer. By definition a mutation in a gene occurs rarely in a population (below 1% of the population under study) while a polymorphism occurs more commonly. Because these polymorphic alleles can act cooperatively and many genes in the same signal transduction pathway can show epistatic relationships, single nucleotide polymorphisms (SNPs) and copy number variations (CNVs) can have observable impact upon the incidence of a cancer in a defined population, the age of onset of a cancer, the response to treatment, the frequency of relapse, and the overall survival of a patient population. Thus in addition to inherited mutations, SNPs and CNVs in a population provide a genetic background that can influence the cancer cells harboring the inherited and somatic mutations that arise and cause a tumor. The phenotypes observed in people with inherited mutations in cancer causing genes are an increased incidence of cancers in a family or population and an earlier age of onset of a cancer than observed in the total population. Mutations in the p53 gene show this pattern and in addition multiple independent cancers in the same individual can be observed [3, 12]. Inherited mutations also often produce a limited set or tissue type of cancer such as BRCA1 or BRCA2 with breast and ovarian tumors [3]. It is thought that the BRCA1 and BRCA2 proteins function in many tissues to repair DNA damage, so the limited cancer causation to breast and ovary remains a mystery. Indeed all of the tumor suppressor genes demonstrate a tissue preference in the tumors they cause when they function as inherited alleles but somatic mutations in those same genes are often found in a much wider group of cancers of different tissues [40]. SNPs and CNVs will likely also have limited tissue impact upon cancers and like inherited mutations, functioning throughout development and life, can have cumulative impact over a lifetime. 

The possible role of a SNP or an CNV in cancer is usually demonstrated by an association study correlating the presence of an “at-risk” allele with the incidence of a cancer or a related phenotype. This is fundamentally a statistical argument that provides correlation, not causality. The situation gets better if the at-risk allele can be shown, in vitro or in vivo, to have a different level or activity that could lead to the population wide phenotype. Examples of these correlations are now being demonstrated for results from GWAS and are taking into account tumor subtypes [41]. Thus molecular and cellular studies can provide an important rational for the population study results. In some cases it may also be possible to model the at-risk allele in another genetic system, such as a mouse carrying the alternate human alleles in the orthologous gene of the mouse. One would then explore the phenotypes observed in humans using such a mouse model. In this way it may be possible to move from correlation to causality. In the process of these studies one may learn about the details of the properties of an SNP or CNV that enlightens the population studies. A very good example of this is an SNP, SNP309, in the first intron of the MDM2 gene in humans. The MDM2 protein is a ubiquitin ligase which negatively regulates p53 levels in a cell by polyubiquitination of the p53 protein followed by its degradation. Thus MDM2 levels and activity in a cell regulate the p53 protein levels in a cell. SNP309 in the MDM2 gene comes in two forms, a G-allele and a T-allele. The first intron of the MDM2 gene contains sites for transcription factors that regulate the levels of the MDM2 mRNA. The G-allele binds a transcription factor, Sp-1, better than the T-allele [42]. Ten base pairs away from this Sp-1 site is an ER binding site and the Sp-1 and ER transcription factors can interact so that the highest levels of MDM2 mRNA and protein are produced in cells that are G/G homozygotes and ER+ and exposed to estrogen as shown in breast cancer cells in culture. Indeed the association of the G-allele of SNP 309 with an early age of onset of a cancer is most commonly observed in premenopausal women with ER+ tumors [43]. Thus if one analyzes all women with breast cancers for an association with the presence of the “at-risk” allele of this SNP the statistical test for an association commonly fails. Only when the association is tested with premenopausal females with ER+ tumors can a clear association be found. This is a good example of understanding the biology and genetics before one undertakes large association studies. 

The human genome of any individual contains about three million SNPs that distinguish that person from another. In the population of humans there are an estimated fifty million SNPs. Most of these differences have no detectable phenotype. Because of this, large genome wide scans (GWAS) of SNPs now employ a million SNPs to test for an association with a disease [44]. This is clearly an exercise in multiple hypothesis testing and so one requires very large populations of cases (and controls) and a statistical significance (a type 1 error rate) that provides a *P* = 10^−7^ value. Even then the number of false positives can be large and so repeated independent studies are required to refine the truly significant associations. This is in part why many SNP associations with cancers have been so poorly reproducible. Small study populations will often give lots of false positives not observed in independent repeat studies. In case-control studies where one is examining the different allele frequencies in a case and a control group there is presently no mathematical test to prove that these two populations are equivalent. Allele frequencies can differ in racial groups or other populations and while it is easy to control for some parameters we do not know all of the variables. In spite of these difficulties several recent publications employing GWAS approaches with large populations have been reported for associations with breast cancers, colorectal cancers, lung cancers, melanomas and prostate cancers [17, 22, 24, 25, 45–51]. 

**Figure 1:**
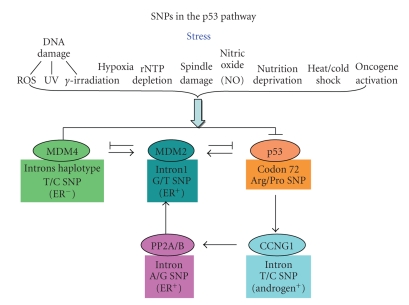


A different approach to uncovering active SNPs with associations to cancers is to examine a small number of candidate genes for the presence of SNPs that impact upon a phenotype. The criticism of this approach is that novel genes and SNPs that impact upon a cancer will not be discovered by this approach. Rather it is a chance to delve deeper into the diversity and properties of a gene, its protein and its phenotypes in a population. The most likely candidate genes that have functional SNPs are the ones that have mutations in some cancers or provide an inherited basis for cancer when they are mutated. Because SNPs are expected to be less deleterious than a mutation which inactivates or fully activates a function, it is helpful to look for candidate SNPs in genes that demonstrate haploinsufficiency. This encompasses those genes that have a cancer-related phenotype when an individual has only one wild type allele, and presumably half the activity and level of a protein. Interestingly the p53 gene, MDM2 gene, and the MDM4 gene (a second negative regulator of p53 that acts upon MDM2) are all haploinsufficient genes in mice and p53 is haploinsufficient in humans (there is presently no test for MDM2 or MDM4 haploinsufficiency in humans) [52, 53]. These three proteins make up the core of p53 regulatory activities in a cell (see Figure 1). There is a great deal of evidence demonstrating that the levels and/or activities of these proteins in a cell are tightly controlled by extensive feedback loops and that small changes in these proteins have phenotypes that are readily observed. 

Another way to look for SNPs that have biological activity is to examine whether a mutant allele or a polymorphic allele is under negative or positive selection in a population. If that is the case then that allele must have a biological activity that impacts upon the organism. There are now a growing number of methods to look for regions of a genetic locus under positive or negative selection. Selection pressures in humans commonly result from genes that contribute to resistance to infectious diseases (about 20% of human cancers are caused by or associated with viruses), optimal use of nutritional opportunities (the IGF-PI3K-PTEN-mTOR pathways help to regulate this), or the highest levels of fecundity, leaving more offspring in a population (the p53 pathway can participate in this and is discussed below). Employing information theory-entropy based methods Atwal et al.have suggested that some alleles of MDM2 and MDM4 are under positive selective pressures in Caucasian populations [54, 55]. Based on identification of selected loci in MDM4, studies have now demonstrated associations between MDM4 SNP loci with risk of breast and ovarian cancers as well as age of onset of ovarian cancers and hormone receptor negative breast cancers [55, 56].

There is also evidence that an allele in the coding region of the p53 protein may also be under positive selective pressure in Caucasian populations. This SNP at codon 72 in the p53 protein (out of 393 amino acids) either encodes an arginine (Arg) or a proline (Pro) residue. The Pro-allele is the ancestral form and Africans near the equator have very high levels of the Pro-allele. As populations move to northern latitudes in Europe (Caucasians) and in Asia (Asians) there is an increasing frequency of the Arg-allele reaching 75–85% in Scandinavia. One explanation for this distribution comes from the observation that p53 induces the synthesis of pro-opiomelanocortin which regulates the tanning response. This could be thought of as a protective mechanism for light-skinned populations or helping to protect individuals of lighter skin color which was developed to enhance the production of vitamin D in northern climates. In a recent study in China, a correlation was found that implicates both temperature and ultra-violet light sources as the driving forces upon selection of the p53 Arg/Pro and SNP309 polymorphisms [57].

There is a growing body of evidence that the newly formed and selected Arg-allele in Caucasian and Asian populations has quite different properties then the Pro-allele. Cells in culture with the Arg-allele transcribe several pro-apoptotic genes at higher rates than the same cell lines with Pro-alleles. Several studies have demonstrated that cells with the Arg-allele undergo higher frequencies of apoptosis than the same cells with Pro-alleles. A deletion of the p53 protein proline rich domain, in which the Arg/Pro polymorphism resides, reduces the efficiency of apoptosis by that mutant p53 protein. These studies demonstrate at the cellular and molecular level that functional differences exist between these two alleles of the p53 gene [58–61].

Perhaps the best explanation for the selection of the p53 Arg allele in Caucasian populations is the observation that the cytokine leukemia inhibitory factor (LIF) is regulated at the transcriptional level by p53 and two times more LIF is produced in cells by the Arg-allele than the Pro-allele of p53 [62]. p53-mediated production of LIF in the uterus is required for implantation of mouse embryos after fertilization (LIF is also produced in humans for implantation) and so both p53 and LIF are required for high levels of fecundity [63]. Interestingly, the frequency of the p53 Pro-allele is quite enriched in women who are at an in vitro fertilization clinic and demonstrate lower levels of implantation of fertilized eggs [62]. This observation may explain the selective pressures on these alleles in Caucasians. Obviously there are not similar fertility difficulties in Africans with the Pro-allele, suggesting that the genetic background (other alleles in genes in the p53 pathway) is an important factor for this phenotype. Poor fertility was observed in p53 knockout mice and varied in different genetic backgrounds [63]. Some of the compensating MDM2 and MDM4 alleles are also under selection pressures in Caucasian populations. It has been these types of studies that have identified functional SNPs that can now be tested for their activities and associations with specific cancers. 

## 4. The p53 Pathway and Cancer Prevention

The p53 protein and its signal transduction pathway respond to a wide variety of stresses and act as a fidelity check point preventing mistakes leading to high mutation rates. Cellular stresses such as DNA damage, telomere shortening, hypoxia, nutrient deprivation, an interruption of ribosome biogenesis, errors in proper mitotic spindle functions, or even the mutational activation of selected oncogenes (myc, Ras) can activate the p53 protein so that it becomes an efficient transcription factor for selected genes. A wide variety of protein kinases, histone methylases, ubiquitin ligases, and so forth participate in detecting these stress signals and modifying the p53, MDM2, or MDM4 proteins. This results in shutting down the MDM2/4 negative regulation of p53, an increased half-life, and an increased concentration of the p53 protein in the cell (Figure 1). Higher levels of a modified p53 protein then give rise to a transcriptional response of p53 regulated genes. The most common outcomes of this signal transduction pathway are apoptosis, cellular senescence, or cell cycle arrest. Because these stresses upon a cell can cause a very high error rate in both DNA replication and cell division, p53 blocks progression through the cell cycle or eliminates clones that contain mutational events. In this way p53 acts as a tumor suppressor gene over a lifetime of stressful events. The presence of only one wild type p53 allele in mice or humans (Li-Fraumeni syndrome) leads to an early onset of tumors compared to the wild type population and often leads to multiple independent tumors in an individual with almost a one hundred percent penetrance [64, 65]. Four independent studies have now shown that the G-allele (at-risk allele) of SNP309 in the MDM2 gene, which raises the levels of this mRNA and protein inhibiting p53 activity, lowers the age of onset of tumors and increases the number of independent tumors observed in individuals with Li-Fraumeni syndrome [42, 66–68]. Those individuals with Li-Fraumeni syndrome who do not have a p53 mutation (patients with a high frequency of tumors due to an unrelated mutation) are not affected by the SNP309 G-allele demonstrating the specificity and epistatic relationship between MDM2 and p53. Li-Fraumeni patients with a p53 mutation also have more rapid telomere erosion, demonstrating the role of p53 and SNP309 in this process [67]. It is well known that p53 senses the loss of telemetric DNA and will stop cell division or cause cellular apoptosis. One study examining both the p53 Arg/Pro and SNP309 polymorphisms suggested that the p53 Pro-allele can have an impact upon the age of onset of cancers (earlier) and survival (poorer) in patients with Li-Fraumeni syndrome and SNP309 can make the situation worse, but the number of patients with both genotypes was too small to obtain a statistically confident result [68]. 

## 5. SNPs in the p53 Pathway Associated with Breast and Ovarian Cancers

The literature examining the association of SNPs in genes in the p53 pathway is fraught with contradictions. Undoubtedly, this comes about for several reasons. First, studies involving small population sizes do not necessarily provide adequate statistical power. Second, studies may fail to stratify populations into groups that reflect the biology and clinical impacts of a cancer. For example, MDM2 SNP309, which acts preferentially in premenopausal females with ER+ tumors, is a smaller group in the total cohort. Third, there is a failure to understand that SNPs in different genes may have very different phenotypes in the context of a cancer (tissue specificity, the age of onset phenotype, etc.). Finally, there is a tendency to make comparisons of cases and controls that are not biologically or genetically equivalent. 

At present, the structure of many association studies leads to false positives and negatives as well as uncovering an occasional functional SNP. For example, a very large population of women with breast cancer was analyzed in a GWAS but the patients were not stratified by ER status or for any of the clear differences between different types of breast cancers [22]. Clearly, this will dilute any signal that comes from just one of these cancer types. In addition, when SNPs in tumor suppressor genes or oncogenes are under study it may be the case that a mutation in that gene will eliminate the SNP from being identified or all SNPs in genes epistatic to the mutated gene may no longer score in the association study. Because of these difficulties we must rely upon the independent replication of results as well as a functional explanation for how an SNP is acting to uncover an association. In a formal large meta-analysis of published results from the literaturevan Heemst et al.[69] studied the impact of p53 Pro/Pro and Arg/Arg polymorphisms upon the frequency of developing cancers and upon the longevity of the population under study. They found that individuals with a Pro/Pro genotype had an increased risk of developing a cancer over their lifetimes when compared to individuals with an Arg/Arg genotype. In a prospective study of individuals 85 years and older, carried out with 1226 people over a ten-year period, they found that people with the Pro/Pro genotype had a 2.45 increased proportional mortality from cancer (*P* = .007). But this group also showed a longer longevity (a 41% increased survival in the population, *P* = .032). One interpretation of this result is that the Arg/Arg genotype has a higher apoptotic rate in response to stress and so protects against cancer better, but also kills stem cells more efficiently over a lifetime, reducing longevity [69]. This suggests that studying older patients may reveal a phenotype in this p53 SNP because it acts over a lifetime to protect the host from stresses. By the same token, older mice show declines in p53 activities with age and this lower level of p53 responses could uncover a phenotype at older ages that is too robust to measure in younger groups [70]. If this interpretation is correct one can see why the p53 Arg/Pro SNP has given rise to such contradictory responses when the ages of the case and control groups are not taken into account. 

Similarly, studies with MDM2 SNP309 have produced contradictory associations with cancers. Some of this has to do with mixing both males and females into the cohort under study (SNP309 is regulated by the ER), some of this has to do with a failure to separate ER+ and ER− tumors in the analysis, and some studies just choose the wrong phenotype or cohort to measure. For example, the observation that the G-allele (the at-risk allele) of SNP309 is associated with an earlier age of onset in a variety of cancers has now been reproduced by many independent groups employing soft tissue sarcomas [43], lymphomas [43], leukemia [71], melanomas [72], head neck [73] and oral squamous cell carcinomas [74], gastric cancer [75], colon cancers [76, 77], lung cancers [78–80], endometrial cancer [81, 82], bladder cancers [83, 84], glioblastoma [85], neuroblastoma [86], and both breast cancers [43] and ovarian [87] cancers. In a study of lung cancers, Lind and her colleagues [78] found that G/G homozygotes had a 1.62 odds ratio of developing cancer compared with T/T homozygotes. When only females in the study were considered the G/G to T/T odds ratio was 4.06. This is the same result observed in an independent study with large diffuse B-cell lymphomas where the G-allele of SNP309 was associated with cancer only in premenopausal females and not in postmenopausal females nor in men [43]. 

In a third recent study of melanomas, a similar association was found only in premenopausal females [72]. This pattern suggests that active estrogen receptors are present in a large number of tumors and can affect the outcome of the disease. This set of genetic observations opens up possible new routes for therapy with these tumors. In another large study with lung cancer patients carried out in China by Zhang et al.[80] they demonstrated an odds ratio of 1.83 for the G/G over T/T alleles and a 1.47 fold odds ratio for the p53 Pro/Pro over Arg/Arg individuals. Those patients who were G/G and Pro/Pro had an odds ratio of 4.36; whereas those smokers (a mutagenic stress that activates p53) who were Pro/Pro, G/G had an odds ratio of 10.41. Understanding the biology of the signal transduction pathway can permit one to study the relevant variables. Often combinations of SNPs that have an epistatic relationship can provide much more significant results. 

Recently, biologically functional SNPs were detected in the MDM4 gene that appear to be under evolutionary selection pressures and have an impact upon fecundity in females at an IVF clinic [62, 63]. Association studies employing five different patient populations have indicated that selected alleles of these SNPs confer an increased risk for or early onset of breast cancers and ovarian cancers [55, 56]. The ethnic backgrounds of the cohorts under study made a difference in the ability to detect these associations so that once again a genetic background of other SNPs that reside in other genes in the same pathway could play an important role. The minor alleles of these same MDM4 SNPs demonstrated a clear enrichment in their frequencies in women at an IVF clinic who had difficulties with implantation of embryos. These diverse phenotypes suggest a functional consequence of these SNPs that can be selected for or against over recent (Caucasian and Asian) times of human evolution. It will be important to observe replications of these data in a wide variety of cancers. 

Because many of the cancer treatments result in DNA damage and other stresses to a cell, the response (as determined by a combination of SNPs) to treatment and long-term survival could depend upon the combination of alleles in the p53 pathway SNPs. To test this notion association studies will have to assemble large groups of individuals who have experienced a defined cancer and treatment and record the outcomes over many years. Such cohorts are more difficult to assemble but are an important part of this effort. Characterizing the patient and the tumor genome prior to the selection of treatments is a growing concern and would be aided by the use of validated SNPs and mutations. 

## 6. Limitations with SNP Studies

The success of these studies has been in part due to the use of large study samples (usually based around multicenter consortia) and replication data sets that have been designed into the gene discovery algorithm. Although this approach maximizes the identification of possible genes involved in contributing to breast cancers, these studies often give rise to a number of false positive findings due to multiple hypothesis testing with a very large number of SNPs in the GWAS scans. In addition, the GWAS approach tends to optimize the discovery of genes with statistically significant marginal effects. Therefore, it may miss significant genes. First, genes may not be detected whose effects are not significant on the margin but are significant in conjunction with other genes or exposures. Second, genes may not be identified if there is substantial genetic heterogeneity among cases, such that the proportion of individuals in the population whose disease is caused by a gene is so small that its effect is “washed out” in the total sample. Third, GWASs tend to favor large numbers over epidemiologically rigorous study designs. Although large samples are clearly required to detect small effects, and replication/validation of initial results maximizes the chances that reported associations are true positives, it is possible that unmeasured biases due to study design limitations may have resulted in high false negatives. 

Likewise, meta-analyses, used to critically evaluate and statistically combine studies, have been performed for MDM2 SNP309. However, a caveat to such an analysis is that the studies are comparable. Population-specific effects and SNP functionality in independent racial genetic backgrounds may exist and limit the ability to combine heterogeneous study groups. To emphasize this, Shi et al. describe a causative selection of MDM2 SNP309 and p53 Arg72 associated with environmental stresses, that is, cold winter temperatures and UV intensity, wherein the two SNPs are not coselected [57]. This is further supported by two publications describing population-specific differences between African-Americans, Caucasians, and Caucasians of Ashkenazi Jewish descent for both MDM2 SNP309 and MDM4 haplotypes [54, 55]. A combined analysis for SNP309 was presented in Wilkening et al. [88]. Data from eleven breast cancer studies, five colorectal cancer studies, or seven lung cancer studies were each combined for a fixed meta-analysis. Based on their analysis, they concluded that the SNP309 variant did not have an impact on risk or colorectal cancers, but did exhibit increased risk in the homozygous state for lung cancer. They concluded that SNP309 alone has little effect on the risk of common cancers. In reviewing criteria of studies within the analysis, there is significant heterogeneity between study groups. Other studies have also previously concluded that the effects of SNP309 are evident in women but not in men and in hormone receptor-positive breast cancers [43]. Most studies do not differentiate between gender or hormone receptor positive diseases, both of which may dilute any effects. In contrast to the conclusions made by Wilkening et al. [88], those of Hu et al. [89] are that the homozygous variant is associated with increased risk of all types of tumors where tumor type and ethnicity contributed to substantial heterogeneity. The latter publication by Hu et al. [89] describes in detail methods for identifying appropriate studies, data extraction and analysis. The majority of publications fail to reduce heterogeneity in their populations based on molecular markers, gender, and degree of disease heterogeneity. Therefore, a meta-analysis would be limited by these factors and should be interpreted with caution.

The majority of useful and reproducible reports involving SNP associations with breast, ovarian, and endometrial cancers have occurred in the context of candidate gene studies. These studies typically identify loci that are hypothesized to contain genetic variants that may be associated with disease risk. Using this approach, a number of putative susceptibility genes have been identified for these tumor sites that have been validated. Furthermore, candidate gene studies have tended to have used more rigorous study designs, collected useful epidemiological and confounder data, and have detailed information that may define etiologically heterogeneous groups of individuals that may provide a setting in which genes that are not easily detectable in the usual GWAS setting may be found. This comes about because we have a great deal of information about those genes already known to play a role in cancer causation that modifies the questions we ask and the associations we look for in a study. It appears that both the GWAS algorithm and the candidate gene algorithm may have value in identifying susceptibility genes, and these genes may be different because of the strengths and weaknesses of each approach.

## Figures and Tables

**Table 1:** Genetic loci implicated in hereditary, familial, and sporadic breast cancer susceptibility.

High penetrance,	Low penetrance,	Low penetrance,
low frequency	low frequency	High frequency
BRCA1	CHEK2	FGFR2
BRCA2	ATM	LSP1
PTEN	PALB2	MAP3K1
p53	BRIP1	TGFB1
STK11		TOX3
		2q35
		8q
